# The Value of Cryosurgery in Treating a Case of Thoracic Chondrosarcoma

**DOI:** 10.1155/2011/243243

**Published:** 2011-05-10

**Authors:** Kalliopi Alpantaki, George Datsis, Odysseas Zoras, Alkisti Kampouroglou, Ioannis Drositis, George Halkiadakis, Pavlos Katonis

**Affiliations:** ^1^Departments of Orthopaedics and Traumatology, University Hospital of Heraklion, Voutes, Heraklion, 71003 Crete, Greece; ^2^Department of Surgery, University Hospital of Heraklion, Voutes, Heraklion, 71003 Crete, Greece; ^3^Department of Thoracic Surgery, University Hospital of Heraklion, Voutes, Heraklion, 71003 Crete, Greece

## Abstract

Chondrosarcomas of the spine are rare and difficult to treat. In this paper a case of thoracic chondrosarcoma is presented. Chondrosarcomas of the spine are generally smaller, more difficult to excise and are followed by higher local recurrence compared with chondrosarcomas of the peripheral skeleton. The tumor is radio- and chemoresistant, making the surgical treatment of utmost importance. The most important prognostic factor for local control is wide or marginal tumor resection. Our patient was treated in two stages, with total excision of the tumor, using cryosurgery. Liquid nitrogen was used to freeze the damaged tissue at a cellular level and made the excision more efficient.

## 1. Introduction

Chondrosarcoma is the most frequent primary malignant bone tumour. It constitutes a family of malignant tumors in which the cells differentiate into cartilage and may originate from healthy bones (primary) or from chondromas or osteochondromas with sarcomatous degeneration (secondary). Chondrosarcomas are classified into three categories: central, peripheral, and periostal as well as two rare entities: mesenchymal chondrosarcoma and clear-cell chondrosarcoma. Primary chondrosarcoma of the spine is an extremely rare tumor. Only six to ten percent of chondrosarcomas arise from the mobile spine. The maximum incidence of chondrosarcoma is found in adult, male patients with a tumor diameter being generally greater than 5 cm.

Radiographically it is identified as an area of bone destruction and a calcified soft tissue mass. The soft tissue mass may be absent in lesions originating from a vertebral body. Magnetic resonance imaging (MRI) can best demonstrate the extent of the lesion and of the cord compression. Usually chondrosarcoma does not manifest signs of cord compression at early onset [1]. 

Chondrosarcoma of the spine is challenging to manage. The clinical course of chondrosarcomas originating from the spine is usually long because of the slow growth of the tumour [1]. Local recurrence and metastases, resistant to most protocols of radiation therapy and chemotherapy, may occur in more than 10 years after surgical removal. Surgical excision has been advocated as the primary mode of therapy for treating chondrosarcoma. For best results (longer survival, lower rate of recurrence) a more aggressive surgical approach is preferred [1–7]. 

We report a case of thoracic chondrosarcoma, which was treated with total resection, using cryosurgrery. Cryosurgery is found to be effective against a localized disease and solid tumors larger than 1 cm. Liquid nitrogen was used to freeze the damaged tissue at a cellular level and form ice crystals inside the cells of the tumor making the excision more efficient and with lower rates of side effects. More damage to the tumor occurs when blood vessels supplying the diseased tissue freeze.

Such malignant tumors occurring from the spine pose difficult issues in terms of surgical treatment [8, 9]. There is little mentioned in the literature for treating chondrosarcoma of the spine using cryosurgery [10–14]. It is a field that should be reported and studied in depth.

## 2. Case Presentation

A 37-year-old male consulted our department with a 7-month history of mild thoracic spine pain after a fall; in addition to the thoracic spine pain he noticed a painful, slowly growing mass. He had no past medical history, and physical examination was unremarkable except for the presence of a hard and tender thoracic spine mass. His father died of hepatic cancer. Clinical blood examination did not show any abnormality, including alkaline phosphatase.

**Figure 1:**
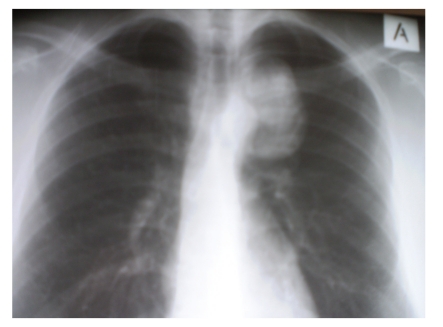
The posteroanterior chest radiograph shows a well-defined radiopaque lesion.

**Figure 2:**
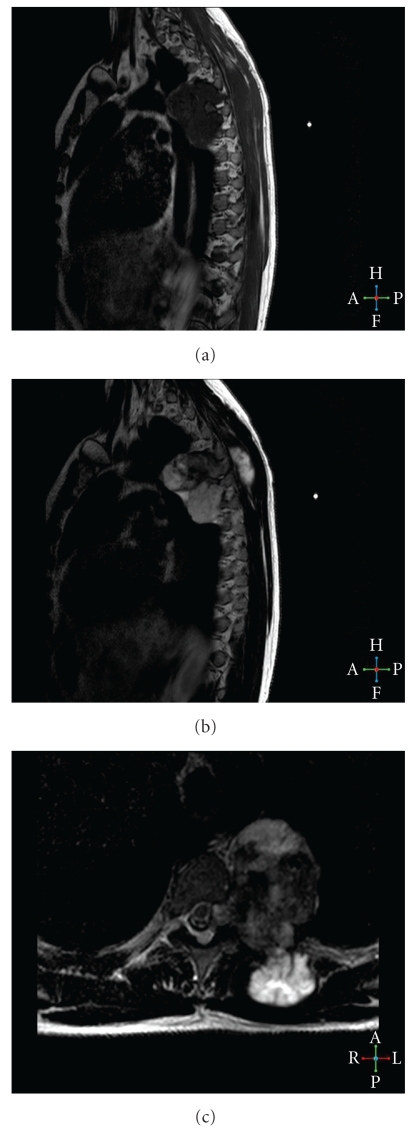
The sagittal T1-w (a) MR image shows a hypointense lobulated lesion. The mass demonstrates heterogeneous but predominantly high signal intensity in the sagittal T2-w. (b) MR image. Note the superficial palpable component of the tumor. (c) The T2 axial view shows how the tumor is positioned next to the ventral structures and its relationship to the spinal cord.

Initial posteroanterior chest radiograph revealed the presence of a left-sided soft tissue mass located at the level of the aortic arch (Figure 1). The lesion's margins and the absence of a positive “silhouette” sign with the mediastinal structures indicated an extrapulmonary and posterior position, a finding that was verified in the lateral chest radiograph. A CT examination was subsequently performed upon request, which showed a predominantly soft tissue tumour with internal areas of mineralisation. The mineralised elements were amorphous, scattered, ring-like, and arcuate-shaped. Rib destruction and infiltration was also noted. Furthermore, widening of the adjacent neural foramina was depicted and an MRI examination was done to clarify this finding. MRI verified neural foramina invasion as well as significant spinal cord compression and contralateral displacement. Despite that fact, the patient remained completely asymptomatic, which was attributed to an indolent and slow growth of the tumour. The mass showed low signal intensity in T1-w images and heterogeneous but predominantly high signal intensity in T2-w images. An associated superficial component of the tumour, which corresponded to the palpable finding, was also better demonstrated on MRI (Figure 2). Mineralised areas that were depicted in the CT examination demonstrated low signal intensity in all pulse sequences. Gadolinium enhanced T1-w fat saturated images showed intense, heterogeneous tumour enhancement. The mass extended from the anterior to the left of the vertebral body at T3–T6 measuring 7.5 × 6 × 5.5 cm and in the left paravertebral space, displacing the aortic arch and thoracic aorta and at T4–T7 in the left epidural space displacing the spinal cord. At T4 the tumor extended posteriorly measuring 4 × 3 × 2 in the paraspinal muscles. Correlation of all the imaging findings with the clinical presentation suggested the diagnosis of a low-grade rib chondrosarcoma, which was verified after a CT-guided biopsy.

**Figure 3:**
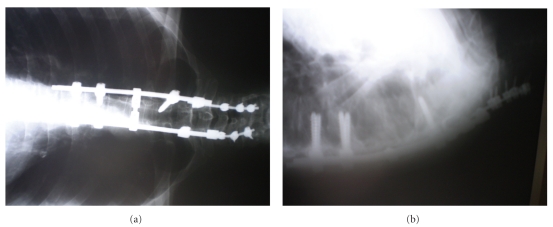
The postoperative plain X-ray.

The surgical procedure was conducted in two stages. Posterior then anterior approaches were planned. For the first stage, the patient was placed prone and an incision was made in the midline extending from C5 to T7. The spinous process and bilateral inferior facets of T3 were removed. The posterior bony elements of T4 and T5 were removed. Instrumentation was placed from C5 to C7 and T3, T6, T7 and fixed with pedicle screws. A combination of Vertex and CD Horizon system (Metronic) of posterolateral fixation was used. Fusion was then accomplished by decorticating the transverse processes and an autograft with bone chips was applied after the exposed dura had been protected (Figure 3). 

**Figure 4:**
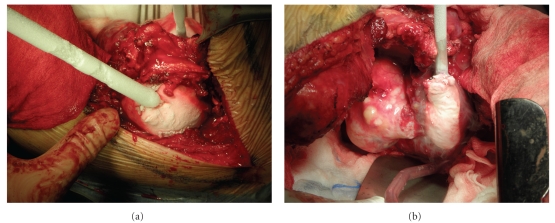
The second stage of the procedure. The tumour had 3 lobes and was treated with liquid nitrogen (−17°C for 15 min/lobe). The equipment used was Candela CS5 (Spembly Medical).

The second stage of the procedure was performed 3 months after the first stage. The patient was placed in a lateral position with his right side down; surgical excision of the 5th allowed a large thoracotomy. The tumour had 3 lobes and was treated with liquid nitrogen (−17°C for 15 min/lobe). The equipment used was Candela CS5 (Spembly Medical) with up to 5 probes, which can be sterilized and reused. We used 3.5 and 10 mm rod-shaped and a plate probe (for small superficial tumors) with liquid N2 (55 psi) which can reach –196°C (tissue temperature is usually 30 degrees higher (Figure 4). Subsequently, the tumour with a part of the 6th rib was resected. Macroscopically, the tumour removal was at least marginal. The wound was copiously irrigated and hemostasis was attained. The wound was closed in multiple layers. 

**Figure 5:**
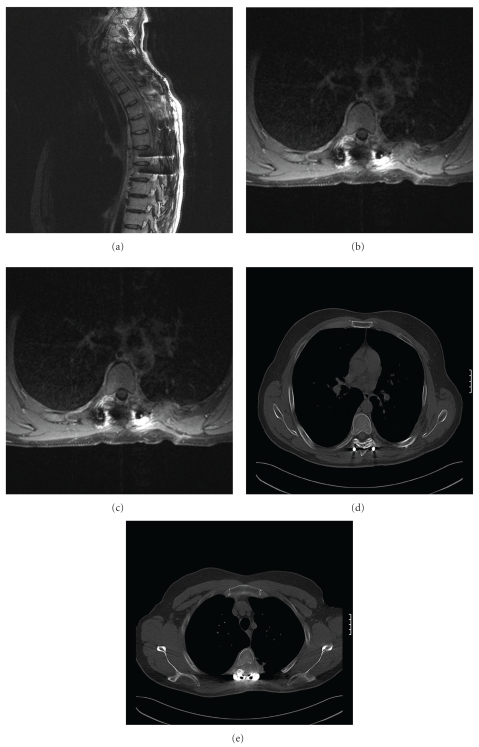
Postoperative MRI (a, b, c) and CT scan (d, e) show no evidence of recurrence six months after the operation.

Histopathology of the resected specimens was grade 1 and grade 2 chondrosarcoma. According to the histopathologic examination of the resected specimen the tumour was marginally resected during the posterior approach and *en bloc* resected with disease-free margins during the anterior operation. Postoperative MRI and CT scan 6 months following the operation confirm the *en bloc* resection of the tumour (Figure 5).

## 3. Discussion

Chondrosarcomas of the spine are rare. Some large series of this particular localisation have been published; however, no specific information on optimal surgical method has been clearly described [15]. Spine chondrosarcoma is generally a low-grade, slowly growing tumor that tends to recur if inadequately managed [2, 5]. Although sarcomas are generally chemosensitive and radiosensitive, the chondrosarcoma subtype is resistant to the known protocols of chemotherapy and also radioresistant, consequently the role of surgical management is preeminent [3, 4]. Techniques developed for “*en bloc*” resection of chondrosarcomas of the limbs have dramatically reduced the incidence of amputations, also improving the prognosis. The application of the same surgical procedures to primary tumors of the spine is technically demanding, requires a deep knowledge of the surgical anatomy of the spine but is often very difficult to achieve. The optimal treatment of spinal chondrosarcoma should be wide or at least marginal surgical excision with tumor-free margins at the resection level. According to the existing literature, “*en bloc* resection” with “wide histologic margins” gives the patient the best chance of survival and can be curative, when it succeeds in removing the whole tumor mass together with a continuous, even thin (marginal margin), shell of healthy tissue [6, 8, 16–18]. 

Since the role of the extent of surgical resection appears to have such an impact on recurrence rate and time of tumor recurrence, the best surgical technique should be used, to offer optimal results [19]. Our results suggest that cryosurgery reduces bleeding. At the end of the first stage of the surgical procedure when no cryosurgery was applied bleeding was estimated at 1.51, in comparison to 75 mL at the second approach with the use of cryosurgery. Our results suggest that the use of cryotherapy improves the surgical technique and offers the best chance for a marginal resection of the tumor. Liquid nitrogen was used to freeze the damaged tissue at a cellular level and form ice crystals inside the cells of the tumor, making the excision more efficient. Careful preoperative planning for surgical tumor removal is mandatory. In addition, literature suggests that cryosurgery reduces the risk of scattering of malignant cells and tissue during the surgical procedure [13, 14].

Although cryosurgery is found to be effective for use against localized disease and solid tumors larger than 1 cm, little is mentioned in the literature concerning the use of liquid nitrogen in the treatment of spine chondrosarcoma [10–14]. It is a field that should be reported and studied in depth.
